# The Antibiotic Efflux Protein TolC Is a Highly Evolvable Target under Colicin E1 or TLS Phage Selection

**DOI:** 10.1093/molbev/msab190

**Published:** 2021-06-27

**Authors:** Yusuf Talha Tamer, Ilona Gaszek, Marinelle Rodrigues, Fatma Sevde Coskun, Michael Farid, Andrew Y Koh, William Russ, Erdal Toprak

**Affiliations:** 1 Green Center for Systems Biology, University of Texas Southwestern Medical Center, Dallas, TX, USA; 2 Department of Immunology, University of Texas Southwestern Medical Center, Dallas, TX, USA; 3 Department of Biomedical Engineering, Johns Hopkins University, Baltimore, MD, USA; 4 Department of Pediatrics, University of Texas Southwestern Medical Center, Dallas, TX, USA; 5 Department of Microbiology, University of Texas Southwestern Medical Center, Dallas, TX, USA; 6 Center for Alzheimer’s and Neurodegenerative Diseases, University of Texas Southwestern Medical Center, Dallas, TX, USA; 7 Department of Pharmacology, University of Texas Southwestern Medical Center, Dallas, TX, USA

**Keywords:** systems biology, protein evolution, deep mutational scanning, bacterial evolution, bacteriophages, antibiotic resistance

## Abstract

Bacteriophages and bacterial toxins are promising antibacterial agents to treat infections caused by multidrug-resistant (MDR) bacteria. In fact, bacteriophages have recently been successfully used to treat life-threatening infections caused by MDR bacteria (Schooley RT, Biswas B, Gill JJ, Hernandez-Morales A, Lancaster J, Lessor L, Barr JJ, Reed SL, Rohwer F, Benler S, et al. 2017. Development and use of personalized bacteriophage-based therapeutic cocktails to treat a patient with a disseminated resistant Acinetobacter baumannii infection. Antimicrob Agents Chemother. 61(10); Chan BK, Turner PE, Kim S, Mojibian HR, Elefteriades JA, Narayan D. 2018. Phage treatment of an aortic graft infected with Pseudomonas aeruginosa. Evol Med Public Health. 2018(1):60–66; Petrovic Fabijan A, Lin RCY, Ho J, Maddocks S, Ben Zakour NL, Iredell JR, Westmead Bacteriophage Therapy Team. 2020. Safety of bacteriophage therapy in severe Staphylococcus aureus infection. Nat Microbiol. 5(3):465–472). One potential problem with using these antibacterial agents is the evolution of resistance against them in the long term. Here, we studied the fitness landscape of the Escherichia coli TolC protein, an outer membrane efflux protein that is exploited by a pore forming toxin called colicin E1 and by TLS phage (Pagie L, Hogeweg P. 1999. Colicin diversity: a result of eco-evolutionary dynamics. J Theor Biol. 196(2):251–261; Andersen C, Hughes C, Koronakis V. 2000. Chunnel vision. Export and efflux through bacterial channel-tunnels. EMBO Rep. 1(4):313–318; Koronakis V, Andersen C, Hughes C. 2001. Channel-tunnels. Curr Opin Struct Biol. 11(4):403–407; Czaran TL, Hoekstra RF, Pagie L. 2002. Chemical warfare between microbes promotes biodiversity. Proc Natl Acad Sci U S A. 99(2):786–790; Cascales E, Buchanan SK, Duché D, Kleanthous C, Lloubès R, Postle K, Riley M, Slatin S, Cavard D. 2007. Colicin biology. Microbiol Mol Biol Rev. 71(1):158–229). By systematically assessing the distribution of fitness effects of ∼9,000 single amino acid replacements in TolC using either positive (antibiotics and bile salts) or negative (colicin E1 and TLS phage) selection pressures, we quantified evolvability of the TolC. We demonstrated that the TolC is highly optimized for the efflux of antibiotics and bile salts. In contrast, under colicin E1 and TLS phage selection, TolC sequence is very sensitive to mutations. Finally, we have identified a large set of mutations in TolC that increase resistance of *E. coli* against colicin E1 or TLS phage without changing antibiotic susceptibility of bacterial cells. Our findings suggest that TolC is a highly evolvable target under negative selection which may limit the potential clinical use of bacteriophages and bacterial toxins if evolutionary aspects are not taken into account.

## Introduction 

**Fig. 1.**
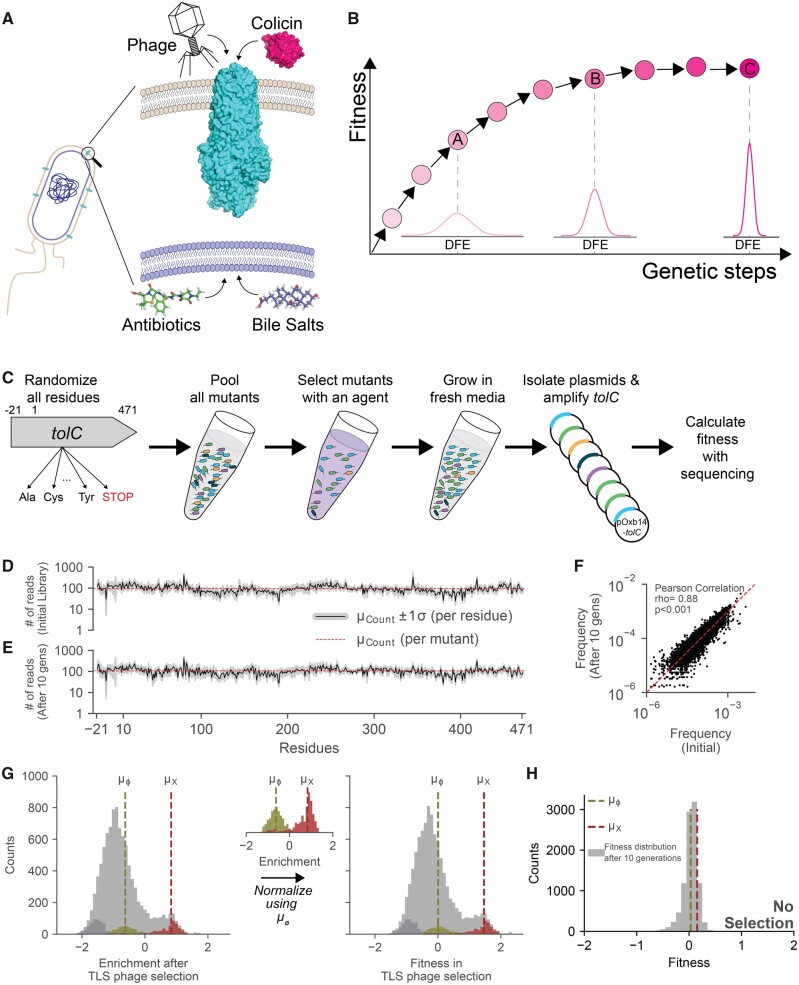
Creation of TolC mutant library. (*A*) TolC is an outer membrane protein in *Escherichia coli* that is involved in the efflux of antibiotics (piperacillin-tazobactam) and bile salts. TolC protein is also exploited as a receptor by TLS phage and colicin-E1, a bacterial toxin. (*B*) Fisher’s geometric theorem predicts evolvability of biological systems using the widths of distributions of fitness effects (DFE). Large DFEs are indicators of high evolvability. Narrow DFEs are observed when biological systems are robust to genetic perturbations and the narrow width suggests lower potential to evolve. (*C*) Experimental procedure for whole gene saturation mutagenesis and fitness measurements. Deep Mutational Scanning of TolC was done by randomization of all 493 residues (including the signal sequence between residues −22 to −1, except the start codon) to 19 other amino acids and the stop codon. All mutants were pooled together and grown under selection of one of the four agents (piperacillin-tazobactam, bile salts, colicin-E1, or TLS phage). The *tolC* alleles were then harvested after a brief recovery growth in plain growth media, and frequency of mutations were calculated by deep sequencing of the *tolC* alleles. (*D* and *E*) Number of reads for all TolC mutations are plotted before (*D*) and after (*E*) growing the TolC mutant library for ten generations (lower panel) in minimal media. Black line represents the mean (*µ*) number of reads per mutation at each residue. Gray area around the black line shows ±1 SD from the mean. Horizontal dashed red line marks the mean number of reads for all TolC mutations. (*F*) Frequencies of all *tolC* mutations before and after ten generations of growth in minimal media were highly correlated (Pearson, rho= 0.88 and *P* < 0.001). Red diagonal dashed line shows *y* = *x* line. (*G*) We calculated enrichment of each mutation by comparing mutation frequencies with and without selection ($e^{i}=\log_{10}(\frac{f_{\mathrm{selected}}^{i}}{f_{\mathrm{unselected}}^{i}}$); e stands for enrichment and f stands for frequency). Light green histogram represents enrichment values of mutations synonymous to the wild-type (WT) TolC protein sequence. Light red histogram represents mutants with early stop codons mutations. Mean values for the stop codon mutations (*µ*_X_) are represented with the vertical dashed red lines. Dark gray colored histograms on the left shows the mutants that went extinct after the selection. Their fitness values were calculated by assuming that their counts were equal to 0.01 after selection in order to manually separate them from the rest of the mutations. We used average fitness of synonymous WT mutations (*µ*_ø_; dashed vertical green line) for defining relative fitness values of each mutation with respect to the WT TolC sequence ($s^{i}=e^{i}-{<e}^{\mathrm{WT}}>$; *s* stands for fitness). (*H*) Distribution of fitness effects for the TolC mutant library after growth in minimal media (∼10 generations) without any selection.

TolC is an outer membrane protein conserved across Gram-negative bacteria and critical for the protection of bacterial cells against the toxicity of antimicrobial compounds such as antibiotics and bile salts (fig. 1*A*) (Paulsen et al. 1997; Dixon et al. 1999; Johnson and Church 1999; Koronakis et al. 2004). TolC forms a homotrimeric channel, composed of a β-barrel domain that spans the outer membrane and a helical coiled-coil bundle that extends ∼10 nm into the periplasmic space. TolC can transiently partner with the AcrA-AcrB, MacA-MacB, and EmrA-EmrB protein pairs to form different tripartite efflux pump complexes in *E. coli* (Piddock 2006). These efflux pumps render *E. coli* intrinsically resistant to several antibiotics such as β-lactams and macrolides by reducing effective antibiotic concentrations inside the cells (Piddock 2006; Ayhan et al. 2016). TolC is also known to efflux bile salts which are abundant in the mammalian gut (fig. 1*A*) (Hamner et al. 2013; Cremers et al. 2014). Therefore, although *tolC* is typically not considered an essential gene for *E. coli*, the loss of *tolC* is costly in the presence of antibiotics or bile salts (supplementary figs. S1 and S2, Supplementary Material online) (Baba et al. 2006). The loss of *tolC* can also reduce resilience of *E. coli* in nature by impairing secretion of hemolysin and colicin V proteins that *E. coli* cells use to eliminate their competitors (German and Misra 2001; Koronakis 2003, 2004; Higgins et al. 2004). As TolC is exploited by both colicin E1 and the lytic TLS bacteriophage as a receptor (German and Misra 2001), exposure to antibiotics (or bile salts) or colicin E1 (or TLS phage) creates opposing selective forces on maintaining the function of TolC, creating a convoluted fitness landscape where the evolutionary dynamics of TolC is highly unpredictable (German and Misra 2001). 

Mutational robustness is a common characteristic of an evolvable protein. For evolutionary success, a protein must tolerate spontaneous mutations for both survival and functional innovation. In the past decade, there have been many studies systematically assessing the fitness effects of mutations, particularly single amino acid replacements, on the function of proteins. Several of these studies have shown that protein function is robust to most single amino acid replacements (de Visser et al. 2003; Wagner 2005; Bloom et al. 2007; Stiffler et al. 2015). In other cases, however, it was shown that many mutations can significantly deteriorate or even impair protein function (de Visser et al. 2003; Wagner 2005; Bloom et al. 2007; Stiffler et al. 2015). As was originally proposed by Fisher, this problem gets even more complex because of the pleiotropic effects of mutations that can improve or worsen multiple traits simultaneously (Fisher 1930). To date, most studies of the evolutionary dynamics of proteins have focused on the selection pressure imposed by a single, specific growth condition, although the natural process often involves multiple, potentially opposing selection pressures (Darwin 1859; Fisher 1930; Losos et al. 2013). This difference limits our understanding of how functional protein sequences have adapted to environmental fluctuations.

According to Fisher’s fundamental theorem, the distribution of fitness effects (DFE) for mutations can be used as a metric for protein evolvability (Fisher 1930; Orr 2006, 2009). Using this theorem, we have previously shown that the width of the DFE is a good predictor for the rate of evolution of antibiotic resistance (Chevereau et al. 2015). If a biological system is highly fit in an environment and robust to genetic perturbations, mutations are expected to have small fitness effects yielding a narrow DFE, centered around neutrality (fig. 1*B*, point C). However, if the biological system has low fitness and is sensitive to genetic perturbations, mutations are expected to have larger fitness effects and hence the DFE will be wider (fig. 1*B*, points A and B). Of note, in figure 1*B*, we use normal distributions with varying widths to represent the DFEs, but realistically there is no way of predicting the shapes of these distributions and there may be outlier groups of beneficial or deleterious mutations.

In this study, we systematically explore the evolvability of the efflux protein TolC using a saturation mutagenesis library which contains all possible amino acid replacements for each position of the TolC protein (barring the start codon). Utilizing both positive and negative selection, we performed a deep-sequencing based fitness assay to quantify the fitness landscape of TolC. We systematically assessed the DFEs of ∼9,000 single amino acid replacements in TolC under antibiotics (piperacillin-tazobactam), bile salts, colicin E1, or TLS-phage selection. We demonstrated that TolC is highly optimized for the efflux of antibiotics and bile salts. In contrast, under colicin E1 and TLS phage selection, we found that the TolC sequence is very sensitive to point mutations. Our findings are consistent with previous studies that identified mutations in the *tolC* allele that conferred resistance to colicin E1 and other phages without compromising antibiotic efflux of TolC (German and Misra 2001; Vakharia et al. 2001; Burmeister et al. 2020). This observation is important in the context of public health where agents such as bacteriophage and bacterial toxins are favorably viewed as promising alternatives to antibiotic therapy.

## Results

### Creation of a TolC Mutant Library

We measured the evolvability of TolC by quantifying DFEs of all possible single amino acid replacements in the presence of four physiological stress factors: antibiotics (piperacillin-tazobactam), bile salts, colicin E1, and TLS phage (fig. 1*C*–*H*). First, we generated a *tolC* deletion strain (*E. coli*-Δ*tolC*, see Materials and Methods) which became more sensitive to both antibiotics and bile salts relative to its wild-type (WT) parent strain (BW25113) (supplementary figs. S1 and S2, Supplementary Material online) (Datsenko and Wanner 2000; Baba et al. 2006). The *tolC* deletion strain was also more resistant to both colicin E1 and TLS phage relative to its WT parent (supplementary fig. S1, Supplementary Material online). We reintroduced the *tolC* gene into this strain using a plasmid that has a constitutively active promoter (pSF-OXB14, Oxford Genetics) and rescued both the antibiotic and bile salt resistance and the colicin E1 and TLS phage sensitivity of the *E. coli*-Δ*tolC* strain (supplementary figs. S1, S2, and S4, Supplementary Material online). We mutated all residues except the start codon (471 residues in the mature TolC protein, and the 21 residue-long signal peptide) of TolC and generated a pool of ∼9,841 (492 sites×20 aa and a stop codon) mutants (fig. 1*C* and supplementary fig. S3, Supplementary Material online). We cloned the mutated *tolC* genes into the pSF-OXB14 plasmid and then transformed the *E. coli*-Δ*tolC* strain with this pool of plasmids carrying mutated *tolC* genes. We randomly selected 30 amino acid positions from our library and using Sanger sequencing, confirmed that all 30 of the mutated sites were randomized and these *tolC* alleles did not have unintended mutations at other sites (supplementary fig. S3, Supplementary Material online). For amplicon sequencing, we pooled mutants into five sublibraries and carried out parallel selection and sequencing experiments (see Materials and Methods). We deep-sequenced the *tolC* genes in each sublibrary by utilizing the Illumina MiSeq platform and verified that 98.9% of possible amino acid replacements in the mutant library yielded at least ten counts when sequenced (fig. 1*D* and *E*), with ∼1,800 reads per residue or an average of ∼90 reads per amino acid replacement (fig. 1*D* and *E*). We also confirmed that frequencies of the mutations in the *tolC* library did not change significantly when the library was grown in growth media without selection (fig. 1*F*, ρ = 0.88, *P* < 0.001, Pearson correlation).

### Systematic Quantification for Fitness Effects of TolC Mutations under Positive or Negative Selection

We measured fitness effects of TolC mutations under selection using a liquid-based assay (fig. 1*C*). In brief, we grew mutant libraries in growth media to saturation, diluted them to an OD600 of 0.001, and then grew these cultures in the presence of one of the four selection factors for 3 h. Cells were then washed and grown in nonselective media for 6 h. Finally, we harvested plasmids carrying *tolC* mutants and performed amplicon sequencing to count the surviving *tolC* variants (see Materials and Methods). Of note, all concentrations used in these assays were above the minimum concentrations sufficient to kill WT *E. coli*, except the bile salts. The maximum soluble amount (50 mg/ml) of bile salts in our selection experiments inhibited growth of the *E. coli*-Δ*tolC* strain but not the WT *E. coli* strain (supplementary fig. S2, Supplementary Material online). The duration of selection and recovery periods were chosen to maximize the dynamic range of the measurements and to minimize the chances of losing some alleles during plasmid harvesting (see Materials and Methods and supplementary figs. S1 and S4, Supplementary Material online). More specifically, we used a duration of 3 h for selection (supplementary fig. S1, Supplementary Material online, vertical gray dashed line) in order to maximize the fitness difference between the WT *E. coli* and *E. coli*:Δ*tolC* (supplementary fig. S4, Supplementary Material online). We deliberately avoided longer incubation times because of our concerns regarding colicin E1 stability in media and potential phage replication events during the selection process. A control experiment with no selection was always performed in parallel, to decouple fitness effects due to growth defects.

For calculating fitness, we determined the enrichment of each mutation by comparing mutation frequencies with and without selection ($e^{i}=\log_{10}(\frac{f_{\mathrm{selected}}^{i}}{f_{\mathrm{unselected}}^{i}}$), where e represents the enrichment, and f the frequency of mutation i, fig. 1*G*). We used the average enrichment of synonymous WT mutations ($<e^{ø}>$; ϕ represents synonymous WT sequences and <> indicates average value) as a reference point for defining relative fitness values (*s*) of each mutation with respect to the WT TolC sequence ($s^{i}=e^{i}-{<e}^{ø}>$; fig. 1*G*, green bins). We also compared the fitness effects of early stop codons (fig. 1*G*, red bins) with the *E. coli*-Δ*tolC* strain supplemented with the *tolC* gene (fig. 1*G*, green bins) and confirmed that the results we obtained using our sequencing-based assay qualitatively matched our observations in batch culture (supplementary fig. S4, Supplementary Material online). By comparing the enrichments of mutations in the absence of selection (see Materials and Methods) relative to the frequencies of mutations in the library before any growth or selection, we confirmed that TolC mutations did not have significant fitness effects in the absence of selection (fig. 1*F* and *H*).

**Fig. 2.**
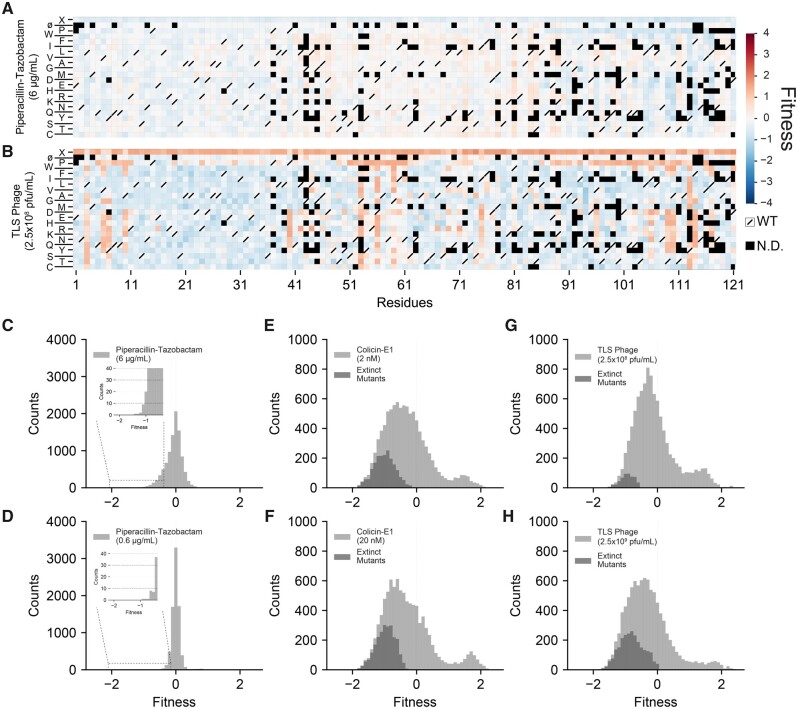
Mutant library selection using piperacillin-tazobactam and TLS phage. Heatmaps summarizing a subset of fitness values of TolC mutations under selection of (*A*) Piperacillin-tazobactam (6 µg/ml) and (*B*) TLS Phage (2.5×10^8^ pfu/ml). Columns within each matrix represent the TolC residues (from residues 1 to 121), and columns represent mutations to other codons causing synonymous(ø), nonsynonymous, or nonsense (X) mutations. Protein sequence of the wild-type TolC is represented by white pixels with cross on them. Mutants with low read counts (threshold=*µ*−1.5*α*) in the initial TolC library or after growth in plain media (untreated) were excluded from fitness calculations and represented by black pixels. All other calculated fitness values were colored from dark blue (increased susceptibility) to white (neutral effects) to dark red (increased resistance). Top row of each heatmap shows the effect of early stop codon mutations (X). Second rows from the top show the effect of silent mutations synonymous to native codon in TolC. Under piperacillin-tazobactam selection (*A*), nonsense mutations increased susceptibility, whereas under TLS phage selection (*B*), nonsense mutations increase resistance. (*C*–*H*) Distribution of fitness effects (DFEs) for different selection agents. For every selection agent, DFEs were calculated under two different selection strengths. DFEs under antibiotic selection are narrow and centered around neutrality (*s* = 0) regardless of the selection strength, with tails extending to the left (increased sensitivity, insets). Under both colicin-E1 and TLS phage selections, DFEs were wide and mean fitness effects of mutations were negative, suggesting that TolC was not robust to mutations under selection to these agents. Under both colicin-E1 and phage selection, many mutations were initially present in the TolC library but went extinct after the selection (dark gray bins). Although it is not possible to calculate fitness in these cases, in order to show them on the histograms, we set their final counts to 1 (pseudocount) and calculated a fitness value such that they are still visible. Under both colicin-E1 and phage selections, DFEs were bimodal with a second peak corresponding to mutations increasing resistance. Means and SDs of light gray histograms are tabulated in supplementary table S1, Supplementary Material online.

Figure 2*A* and *B* shows the fitness effects for a subset of single amino acid replacements in TolC in the presence of antibiotics (6 µg/ml of piperacillin-tazobactam), and TLS phage (2.5×10^8^ pfu/ml). Supplementary figure S5, Supplementary Material online, summarizes the fitness effects of the entire mutation library under all four selection factors. We found that the fitness effects of mutations in the presence of antibiotics or bile salts were mostly neutral (fig. 2*A* and supplementary fig. S5*A* and *B*, Supplementary Material online, white pixels) except a group of mutations increasing sensitivity to antibiotics or bile salts (fig. 2*A* blue pixels, fig. 2*C* and *D* insets). Figure 2*C* and supplementary figure S6, Supplementary Material online, show the corresponding DFEs. When we repeated the same assay using ten times lower dose of antibiotics (0.6 µg/ml, which is still higher than the MIC value of piperacillin-tazobactam for WT *E. coli*, supplementary fig. S2*A*, Supplementary Material online) and bile salts (5 mg/ml), we saw that the DFE in bile salt selection did not change much but the DFE in antibiotics became slightly narrower further verifying the robustness of the TolC sequence under antibiotic selection (fig. 2*D* and supplementary fig. S6, Supplementary Material online). None of the mutations increased resistance to either antibiotics or bile salts suggesting that the *E. coli* TolC sequence is highly optimized for the efflux function as the TolC sequence is mostly insensitive to mutations under these conditions, evident from the corresponding DFEs (fig. 2*C* and *D*; supplementary fig. S6*A* and *B*, Supplementary Material online).

On the contrary, in the presence of colicin E1 or TLS phage selection, the majority of the TolC mutations had large effects on bacterial fitness (fig. 2*B* and supplementary fig. S5*C* and *D*, Supplementary Material online, blue pixels) sometimes making *E. coli* cells more susceptible to colicin E1 or phage-induced death. A subset of mutations increased bacterial resistance to colicin E1 or TLS phage (fig. 2*B*, red pixels). Corresponding DFEs were wider relative to the DFEs in antibiotics and bile salts (fig. 2*E–H* and supplementary table S1, Supplementary Material online). We repeated these measurements using ten times higher concentrations of colicin E1 and TLS phage and showed that the DFEs under these conditions were still wide (fig. 2*F* and *H*). These observations suggested that, under colicin E1 or TLS phage selection (fig. 2*E*–*H*), the *tolC* gene has the potential to evolve resistance. *TolC* resides at a suboptimal fitness state with respect to colicin E1 and TLS, as its sequence is very sensitive to mutations, and both beneficial and deleterious mutations that do not impair efflux are accessible.

### Relationship between Strength of Selection and Fitness Effects

**Fig. 3.**
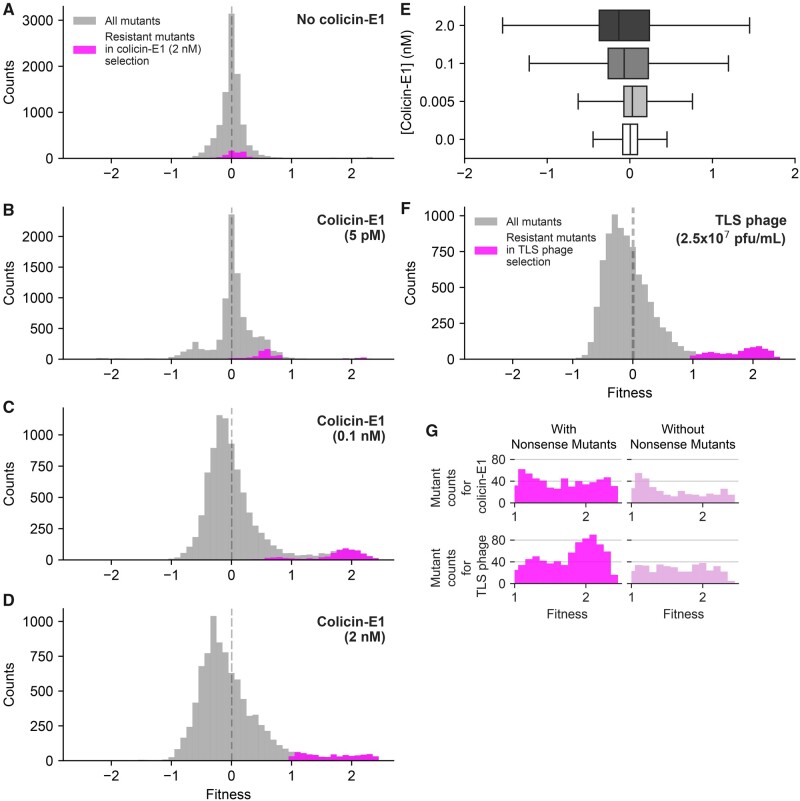
Impact of selection strength on DFE. (*A–D*) Selection strength alters the shape and (*E*) width of the distribution of fitness effects under colicin-E1 selection. We measured fitness effects of TolC mutations by Illumina NovaSeq sequencing platform which yielded ∼100 times more reads per mutation, compared with Illumina MiSeq platform, and increased resolution of our fitness measurements. DFEs for TolC mutations under selection with (*A–D*) increasing concentrations of colicin-E1, and (*F*) TLS phage (2.5×10^7^ pfu/ml). Magenta colored bins in panels (*A*) to (*D*) highlight resistance-conferring mutations that had fitness values larger than 1 (10-fold change in frequency) under selection with 2 nM of colicin-E1. Magenta colored bins in panel F highlight resistance-conferring mutations that had fitness values larger than 1 under phage selection. (*G*) (left) Histograms of all resistance-conferring mutations under colicin-E1 (2 nM, 685 mutations) selection and TLS phage selection (761 mutations). (right) Histograms of all resistance-conferring mutations, excluding stop codon mutations, under colicin-E1 (2 nM, 372 mutations) selection and TLS phage selection (408 mutations).

We measured fitness effects of TolC mutations using different doses of colicin E1 in order to measure the relationship between mutational sensitivity and selection strength. In these experiments, we used increasing concentrations of colicin E1 (0, 5 pM, 0.1 nM, and 2 nM, fig. 3*A*–*E*). In addition, we measured fitness effects of TolC mutations in the presence of TLS phage particles (fig. 3*F*) and bile salts (supplementary fig. S6*C*, Supplementary Material online). These measurements were done using the Illumina NovaSeq platform and yielded nearly 100-fold higher number of reads compared with the MiSeq platform. As the NovaSeq platform provided large number of sequencing reads, we did not observe any extinct mutations and we were able to quantify fitness values with better statistical power. We found that, as the selection strength by colicin E1 increases, the mean values of the DFEs shift to more negative values and the widths of DFEs become larger (SD, fig. 3*A*–*E*). Similarly, the DFE under phage selection was still wide (fig. 3*F*) despite the use of 10-fold fewer phage particles compared with our previous measurements (fig. 2), in agreement with our observations using the MiSeq platform. On the contrary, the DFE under bile salt (5 mg/ml) selection was narrow, similar to the DFE under no selection (supplementary fig. S6, Supplementary Material online). Finally, under both colicin E1 and phage selection, we found that a considerable fraction of TolC mutations were resistance-conferring mutations (fig. 3*A*–*D*, *F*, and *G*, magenta). Almost half of these mutations were early nonsense substitutions (46% for both colicin E1 selection and TLS phage selection) that also induced antibiotic or bile salt sensitivity due to disruption of the efflux machinery. When we excluded stop codon mutations, there were still many (372 mutations spanning 168 residues for colicin E1 selection, 408 mutations spanning 184 residues for TLS phage selection) resistance-conferring mutations suggesting that the TolC sequence was only one mutation away from developing resistance to colicin E1 or TLS phages (fig. 3*G*). Using phages or colicin E1 in combination with antibiotics may potentially reduce the rate of evolution to some extent as early stop codon mutations will be eliminated by the use of antibiotics. However, many resistance conferring mutations will still be available and extended use of phages or colicin E1 in clinical settings may lead to selection of resistant TolC mutants, limiting the success of these therapies in the long term.

**Fig. 4.**
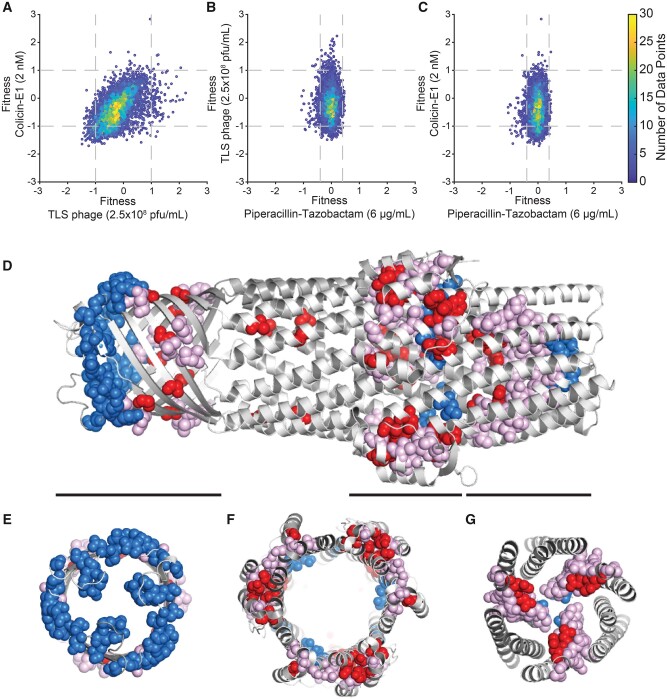
Analysis of mutations from selection experiments. (*A–C*) Comparison of fitness effects of TolC mutations under different selections. These fitness values correspond to consistent values used to plot histograms in figure 2*C*–*H*. Horizontal and vertical dashed lines indicate the significance thresholds (±1.5 SD) for each selection condition, which correspond to ∼10-fold increase or decrease in frequency relative to wild-type TolC (fitness values >+1 and <−1) under colicin-E1 or TLS phage selection. Significance threshold under antibiotic selection were ∼2.5-fold increase or decrease in frequency relative to wild-type TolC (fitness values > +0.4 and < −0.4). (*D*) Side view of the TolC trimer. The 47 most sensitive residues (representing the top 10%) for TLS and colicin E1 are shown in spheres. Positions that show sensitivity to TLS only are colored blue, sensitivity to colicin E1 only in red, and sensitivity to both perturbations in pink. Black bars correspond to slices along the pore axis, shown in (*E–G*) highlighting the three structural regions where sensitive residues are located.

The average fitness effects of TolC mutations under bile salt and antibiotic selection were both very small and weakly correlated (supplementary fig. S6*D*, Supplementary Material online, ρ = 0.28 and *P* < 0.001, Pearson correlation), making it difficult for us to determine whether efflux efficiencies of these molecules by TolC were controlled by similar mechanisms. However, the fitness effects of TolC mutations under phage and colicin E1 selection were broad and significantly correlated (fig. 4*A*, ρ = 0.62 and *P* < 0.001, Pearson correlation) suggesting shared infection mechanisms by these agents. Fitness effects under selection by neither TLS nor colicin E1 showed significant correlation with those measured in the presence of antibiotics (fig. 4*B* and *C*).

### Structural Mapping of Mutation-Sensitive TolC Residues

Comparison of the mean fitness effects of TolC missense mutations in the presence of colicin E1 and TLS phage revealed that resistance to these factors can arise from mutations in three distinct structural regions (fig. 4*D*–*G*), comprising the extracellular surface of the beta-barrel domain, residues near the so-called “equatorial domain,” and residues in the periplasmic pore opening (Koronakis et al. 2000). Although positional sensitivity to TLS and colicin E1 selection showed strong overlap, there were several residues that were more sensitive to one of the two perturbations. Most notably, mutations in a cap of residues on the extracellular surface of the beta-barrel domain caused resistance to TLS phage, but were relatively insensitive to colicin E1 (fig. 4*D* and *E*). Residues near the equatorial domain and in the periplasmic pore exhibited sensitivity under both conditions (fig. 4*F* and *G*). The effects of mutations under different selection factors may provide some insight into the mechanisms by which phage and colicin E1 exploit TolC for entry into the cell.

Looking at individual mutations, rather than the mean effects averaged over each position, provided additional insight. We considered mutations that had fitness effects larger than three SDs from the mean (see Materials and Methods) and excluded early stop codon mutations that were equivalent to loss of the *tolC* gene. For this analysis, we also excluded mutations that did not have consistent fitness effects between experimental replicates (see Materials and Methods). We grouped TolC mutations that passed these criteria as summarized and highlighted mutated residues on the TolC monomer (supplementary fig. S7, Supplementary Material online). Interestingly, many of these residues clustered together suggesting that they might induce similar changes on the TolC structure due to their physical proximity. We found a cluster of residues that result in increased *E. coli* cell resistance (supplementary fig. S7, Supplementary Material online, dark red residues) to both colicin E1 and TLS phage when mutated, without significantly altering antibiotic resistance. Strikingly, there was an independent cluster of mutations, that made *E. coli* cells more sensitive (supplementary fig. S7, Supplementary Material online, dark blue residues) to both colicin E1 and TLS, without significantly changing antibiotic resistance. One plausible explanation is that mutations in the helical coiled-coil bundle extending into the periplasmic space (supplementary fig. S7, Supplementary Material online, highlighted in dark red and dark blue) alter the interactions of TolC with other proteins involved in colicin E1 or phage entry. Although not much is known about TLS entry mechanism into bacterial cells, at least three other proteins are reported to be involved in colicin E1 entry and translocation (TolA, TolQ, and TolR). Mutations in the residues on the beta barrel (supplementary fig. S7, Supplementary Material online, highlighted in dark red) might be increasing resistance by weakening colicin E1 and TLS binding without significantly altering antimicrobial efflux. Mutations in the residues highlighted in cyan (supplementary fig. S7, Supplementary Material online) result in increased resistance to colicin E1 and TLS phage while concurrently increasing sensitivity to antibiotics (piperacillin-tazobactam). This effect can be explained by misfolding of TolC or a possible constriction of the TolC channel that mechanically or electrostatically obstructs the passage of all antibiotics, bile salts, colicin E1, and TLS phage (or viral DNA).

## Discussion

How a protein can navigate new fitness regimes in the presence of multiple stress factors is an open question in evolution. This question becomes even more complex as the ruggedness in a fitness landscape is subject to change with single mutations, the first steps in an adaptive response. To the best of our knowledge, the primary functions of TolC are to efflux antimicrobial molecules and to secrete toxins like hemolysin (Piddock 2006). Its role in colicin E1 and TLS phage uptake is costly for *E. coli*, and these functions most likely evolved as a result of an evolutionary arms race between *E. coli* and its competitors. In the presence of multiple stress factors that are epistatically interacting, adaptive landscapes may become even more rugged. Efflux of antimicrobials and uptake of colicin E1 or phages have opposite fitness effects, and it is unclear how TolC prioritizes these events. For instance, when TolC is occluded with colicin E1, it is uncertain whether antibiotic molecules can pass through the TolC channel (Zakharov et al. 2012, 2016). Similarly, whether colicin E1 can utilize TolC in the presence of antibiotics is unclear, since the antibiotic efflux function involves transient complexes such as the AcrAB-TolC (Blair and Piddock 2009; Blair et al. 2014, 2015) that may preclude colicin E1 translocation. Further, it was previously shown that the translocation of colicin E1 through TolC requires physical interactions between colicin E1 and the three proteins TolA, TolQ, and TolR (Zakharov et al. 2004, 2012, 2016). Whether colicin E1 can physically interact with these Tol proteins while TolC is partnering with other proteins (i.e., AcrA and AcrB) to form efflux pump complexes is not known. Therefore, we anticipate that the evolution of the TolC protein strongly depends on the nature of epistatic interactions between the four stress factors.

TolC forms a homotrimeric outer membrane channel that spans the periplasmic space (Koronakis et al. 2000; Balakrishnan et al. 2001; German and Misra 2001; Koronakis 2003). In order to maintain its efflux activity, TolC has to partner with other efflux proteins such as AcrA and AcrB and maintain the channel opening intact such that the antimicrobial molecules that are pumped by AcrB can pass through. Our findings suggest that the TolC sequence is highly optimized for the efflux function and thus mostly robust to single amino acid replacements. One possible explanation is that of evolutionary optimization of *E. coli* and other Gram-negative bacteria, which reside in environments where bile salts and antimicrobial peptides are abundant (e.g., the mammalian gut) (Cremers et al. 2014). Our data suggest that there are several mutations that can increase resistance to colicin E1 and TLS phage (figs. 3 and 4). Only a handful of these mutations (supplementary fig. S7, Supplementary Material online), however, will likely be selected against in an environment where bacterial populations are simultaneously subjected to antibiotics and colicin E1 (or TLS phage). These mutations can compromise efflux activity by constricting the channel or interfering with the formation of the efflux complexes such as AcrAB-TolC. The majority of mutations that confer phage and colicin resistance are neutral for efflux activity (fig. 4*B* and *C*), and vice versa, indicating that the TolC evolutionary responses to these opposing selection pressures are decoupled. This observation suggests that tradeoffs are relatively rare in TolC evolution and unlikely to play a significant role in the bacterial response to either of these selection factors.

Utilization of bacterial toxins and bacteriophages to fight bacterial infections has been proposed for decades and was successfully used in some life-threatening infections (Chan, Sistrom, et al. 2016; Chan, Turner, et al. 2016). In fact, bacteriophages that bind the *Pseudomonas aeruginosa* OprM, an efflux protein homologous to TolC, were recently used to treat a patient with a life-threatening pan-resistant *P. aeruginosa* infection (Chan et al. 2018). However, it is crucial to develop smart strategies for the clinical use of bacteriophages or toxins to avoid exacerbating the resistance problem, as it was previously shown that bacterial cells can render both bacterial toxins and bacteriophages ineffective (German and Misra 2001; Augustus et al. 2004). *Escherichia coli* can downregulate, mutate, or lose the genes that are involved in colicin E1 entry (i.e., *tolA* or *tolC*) (German and Misra 2001; Ricci and Piddock 2010; Zakharov et al. 2012). Indeed, consistent with our results, several *tolC* mutations (i.e., G43D) as well as complete loss of *tolC* function were previously reported to confer colicin E1 resistance (Masi et al. 2007). Similarly, several *tolC* point mutations (i.e., G281P) as well as the loss of the *tolC* gene were found to confer resistance to TLS phage (German and Misra 2001). Because TolC is also responsible for antibiotic efflux, the nature of these genetic changes suggested a trade-off between resistance to bacteriophages or bacterial toxins and antibiotic susceptibility of pathogenic bacteria (Chan, Sistrom, et al. 2016; Chan, Turner, et al. 2016). However, our findings, along with other recent studies, indicate that bacteria can quickly evade such evolutionary trade-offs through a large number of mutations that increase resistance to phages without changing antibiotic susceptibility (Kortright et al. 2019; Burmeister and Turner 2020; Burmeister et al. 2020). Our observations suggest that if evolutionary aspects are not taken into account, treatments using phages and bacterial toxins are prone to failure in the long term. Our study also provides structural clues for understanding mechanisms of TolC mediated efflux, colicin E1 binding and translocation, and bacterial infection with TLS. Future structural and functional studies investigating interactions of TolC with its partners and the spatiotemporal dynamics of these interactions will help to define strategies for controlling evolutionary outcomes, a key step in addressing problems such as antibiotic and drug resistance.

## Materials and Methods

### Growth Media and Strains

*Escherichia coli* cells were grown at 37 °C in M9 minimal medium (248510, Difco) supplemented with 0.4% glucose (50-99-7, Fisher Scientific) and 0.2% amicase (82514, Sigma). BW25113 WT *E. coli* strain (CGSC No.: 7636) and the **_*Δ*_***tolC732::kan E. coli* strain (CGSC No.: 11430) were obtained from the Coli Genetic Stock Center. Kanamycin resistance marker was removed from the Δ*tolC732::kan E. coli* strain following the protocol in reference (Datsenko and Wanner 2000). This strain is referred as the **_*Δ*_***tolC* strain throughout the manuscript. We whole-genome sequenced both the WT (BW25113) and the **_*Δ*_***tolC E. coli* strains and confirmed that no other mutations besides the *tolC* deletion were present in the **_*Δ*_***tolC* strain.

### Saturation Mutagenesis Assay for the *tolC* Gene

pSF-Oxb14 plasmid was obtained from Oxford Genetics (OGS557, Sigma). This plasmid contained a kanamycin resistance cassette and an Oxb14 constitutively open promoter region. The *tolC* gene was PCR amplified from the BW25113 (WT) strain using 5′-ATTCAAAGGAGGTACCCACCATGA AGAAATTGCTCCCCATTC-3**′** (forward), and 5′-AG AAAT CGATTGTATCAGTCTCAGTTACGGAAAGGGTTATGAC-3′ (reverse) primers. It was then cloned into the pSF-Oxb14 plasmid using the NEBuilder HiFi DNA Assembly Kit (E5520, New England Biolabs), following the protocol provided by the manufacturer. Bold and underlined nucleotides in primer sequences overlap with the plasmid sequence. The integrated *tolC* gene was confirmed to have no mutations by Sanger sequencing.

Whole gene saturation mutagenesis was performed by two PCR reactions individually for each codon in the *tolC* gene, including the first 22 amino acid long signal sequence. First PCR reaction amplified a portion of the *tolC* gene in the pSF-Oxb14-*tolC* plasmid and randomized the targeted codon with a primer that contained a randomized NNS nucleotide sequence (N stands for A, C, G, or T nucleotides and S stands for G or C nucleotides) for the targeted codon (this PCR product is referred as insert). Second PCR reaction amplified the rest of the pSF-Oxb14-*tolC* plasmid (this PCR product is referred as backbone). Our custom software for designing mutagenesis primers is available at https://github.com/ytalhatamer/DMS_PrimerDesignTool. Inserts were cloned onto the backbones using the NEBuilder HiFi DNA Assembly Kit (E5520, New England Biolabs and assembled plasmids were transformed into NEB-5-alpha (C2987, New England Biolabs) cells. Plasmid extraction from these cells was done using Nucleospin Plasmid kit (740588, Macharey-Nagel). As this assay produced libraries per each residue, plasmid concentrations were measured and then equimolar amounts of each library were pooled into five sublibraries for 2×250 bp paired-end MiSeq sequencing (residues 2–110, 90–210, 190–310, 290–410, 390–493) and 12 sublibraries for 2×150 bp paired-end NovaSeq sequencing (1–40, 36–82, 79–124,120–166, 162–208, 204–250, 246–292, 288–334, 330–376, 372–418, 414–460, 456–493). Finally, these pooled sublibraries were transformed into *ΔtolC* strain for selection experiments. All growth and selection assays with the library were done using 50 µg/ml kanamycin in minimal M9 media.

### Colicin E1 Purification

A colicin E1 expression vector with IPTG inducible T7 polymerase promoter was kindly provided by Dr William A. Cramer (Purdue University). Only Colicin E1 was amplified and put back to an empty pET24a plasmid to remove immunity protein. Plasmids were then transformed into BL21-DE3 cells for expression and purification. Cells were grown in TB broth media and colicin E1 was purified first with a size exclusion chromatography. Elutes corresponding to the size of Colicin E1 (∼57 kDa) were further purified using a cation exchange chromatography in Sodium borate buffer with a salt gradient of 0–0.3 M (NaCl). All fractions are collected and analyzed by SDS–PAGE. Elutes with right band sizes pooled and concentrated using Amicon Centrifugal filters with 30 K pore size (UFC803024, Milipore).

### TLS Phage Harvesting

TLS phage strain was kindly provided by Dr Joe Fralick (Texas Tech University). Phage propagation and purification were done following the protocol described in (Fisher 1930). Briefly, overnight grown bacterial cells were diluted hundred times in 100 ml of LB medium with 5 mM CaCl_2_ and incubated 2–3 h till optical density reached 0.4–0.6. Phage particles were added to the culture and the culture was shaken (at 37 °C) until the culture became optically clear. Cell lysates were spun down in 50 ml falcon tubes at 4,000×g and for 20 min. Supernatant was filter sterilized using 0.22 µm filters. Chloroform was added to the filtered phage solution (10% v/v final chloroform concentration) and the solution was vortexed shortly and incubated at room temperature for 10 min. Finally, the phage lysate and chloroform mixture were centrifuged at 4,000×g for 5 min. Supernatant was removed, aliquoted, and stored at 4 °C.

### Selection Assay

We used Piperacillin-Tazobactam (NDC 60505-0688-4, Apotex Corp), bile salts (B8756, Sigma–Aldrich), colicin E1, and TLS phage in selection assays. TolC mutant sub libraries were separately grown overnight in M9 minimal media supplemented with 50 µg/ml kanamycin. These cultures were diluted to the optical density of 0.001 in 10 ml of M9 minimal media supplemented with 50 µg/ml kanamycin (∼5×10^6^ cells). Selection agents were added to each sub library and cultures were incubated at 37 °C for 3 h. All cultures were spun down at 7,000×g for 2 min and pellets were resuspended in fresh M9 minimal media supplemented with 50 µg/ml kanamycin. These cultures were then incubated at 37 °C with shaking for 6 h. Following this step, libraries were centrifuged at 7,000×g for 2 min and pellets were collected for plasmid purification. Different regions of the *tolC* genes were amplified with PCR and indexed using Illumina Index sequences (see Supplementary Material online). These regions were spanning residues 2–110, 90–210, 190–310, 290–410, and 390–493 for 2×250 bp paired-end MiSeq sequencing. For 2×150 bp paired-end NovaSeq sequencing, we amplified and indexed the residues 2–40, 36–82, 79–124,120–166, 162–208, 204–250, 246–292, 288–334, 330–376, 372–418, 414–460, and 456–493.

### Sequence Analysis

Paired ended sequencing reads were first merged using the FLASh tool (Datsenko and Wanner 2000) (customized parameters: -m 40 -M 100). Reads covering primers overlapping with the upstream and downstream of the amplified regions of *tolC* were excluded. Sequence reads were compared with the WT *tolC* sequence and mutations were listed. Sequence reads that had mutations in more than one residue were excluded from the analysis. Synonymous mutations yielding the same amino acid replacement were grouped together. Frequency of each mutation was calculated by dividing number of counts of for that mutation with number of all reads, including alleles with multiple mutations ($f^{i}=\frac{N^{i}}{N^{\mathrm{all}}})$. For calculating fitness, we first determined enrichment of each mutation by comparing mutation frequencies with and without selection ($e^{i}=\log_{10}(\frac{f_{\mathrm{selected}}^{i}}{f_{\mathrm{unselected}}^{i}}$); *e* stands for enrichment and *f* stands for frequency, fig. 1*G*). Since randomized mutations of each residue created traceable mutations synonymous to the WT protein sequence, we were able to use average fitness of synonymous WT mutations for defining relative fitness values of each mutation with respect to the WT TolC sequence ($s^{i}=e^{i}-{<e}^{\mathrm{WT}}>$; *s* stands for fitness, fig. 1*K*, green bins). As a sanity check, we compared the fitness effects of early stop codons with the phenotype of the *E. coli*-Δ*tolC* strain (fig. 1*G*, pink bins) and confirmed that the results we obtained our sequencing-based assay matched our observations in batch culture (supplementary fig. S4, Supplementary Material online). By comparing the enrichments of mutations in the absence of selection relative to the frequencies of mutations in the library before any growth or selection, we confirmed that TolC mutations did not have significant fitness effects in the absence of selection (fig. 1*F* and *H*). Our source code for data analysis is available at https://github.com/ytalhatamer/DMS_DataAnalysis. 

## Supplementary Material

Supplementary data are available at *Molecular Biology and Evolution* online.

## Supplementary Material

msab190_Supplementary_Data
