# Supplementary material for: The Antibiotic Efflux Protein TolC Is a Highly Evolvable Target under Colicin E1 or TLS Phage Selection
Source: Mol Biol Evol. 2021 Jun 27;38(10):4493–504. doi: 10.1093/molbev/msab190 (PMC8476145; doi:10.1093/molbev/msab190)
Supplement: msab190_Supplementary_Data [file msab190_supplementary_data.zip › Tamer_et_al_Supplementary_Figures_Legends.docx]

**Supplementary Figures and Legends**


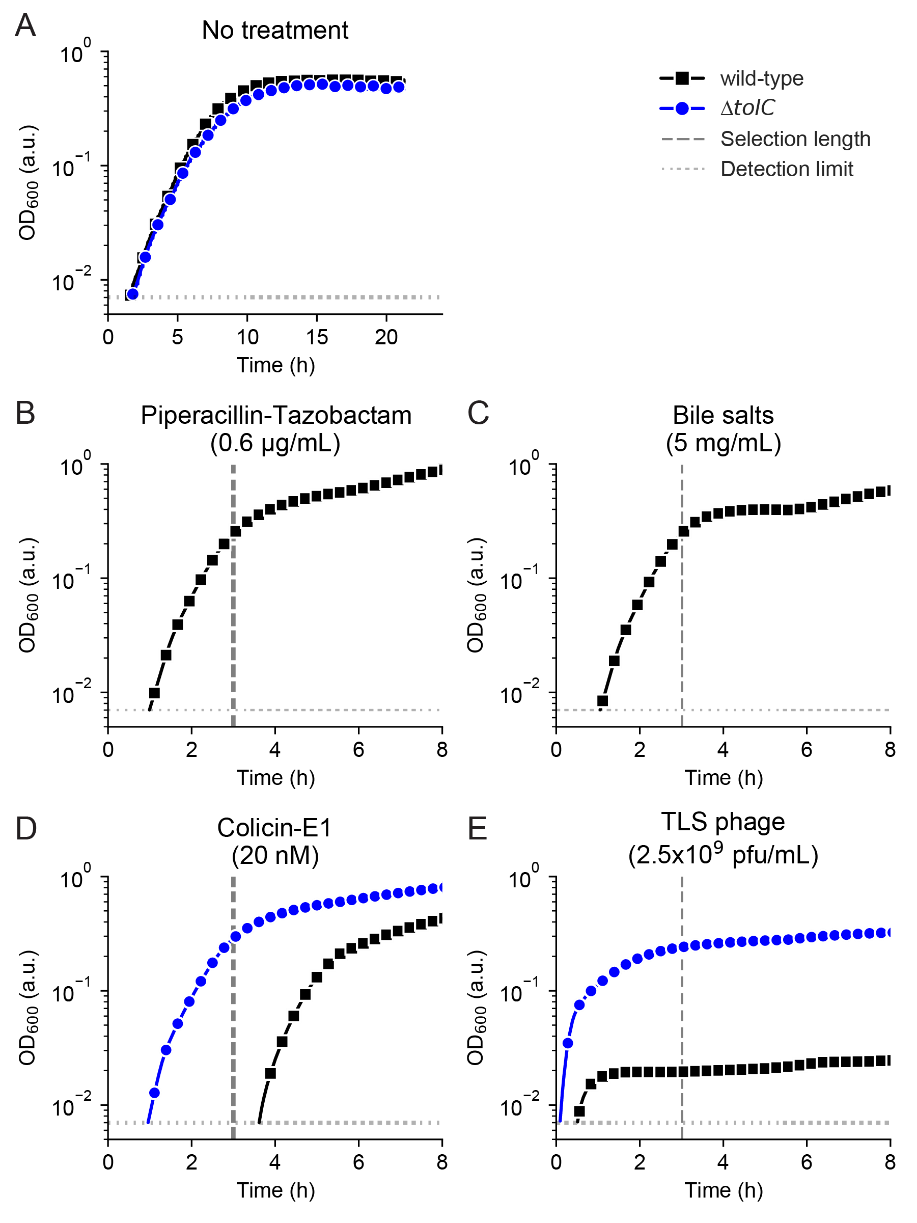


**Figure S1. Deletion of the *tolC* gene is costly under antibiotic and bile salt selection and beneficial under colicin-E1 and TLS Phage selection. (A)** Growth curves of wild-type and *E. coli*:∆t*olC* strains without selection pressure and **(B-E)** 4 different selection factors. We added the selection factors in *E. coli* cultures at the beginning of the experiment and continuously monitored growth curves in a plate reader. Black colored lines represent growth curves of the wild type (BW25113) *E. coli* strain. Blue colored lines represent growth curves of the BW25113 *E. coli* strain with *tolC* gene deletion (∆*tolC*). In our fitness assays, we used a duration of three hours for selection (vertical grey dashed line) in order to maximize the fitness difference between the wild type *E. coli* and *E. coli*:∆*tolC*. Note that *E. coli*:∆*tolC* strain did not show any detectable grow in the presence piperacillin-tazobactam and bile salts (panels B and C). Horizontal gray dotted line represents the detection limit of the spectrophotometer used (OD_600_: 0.007).


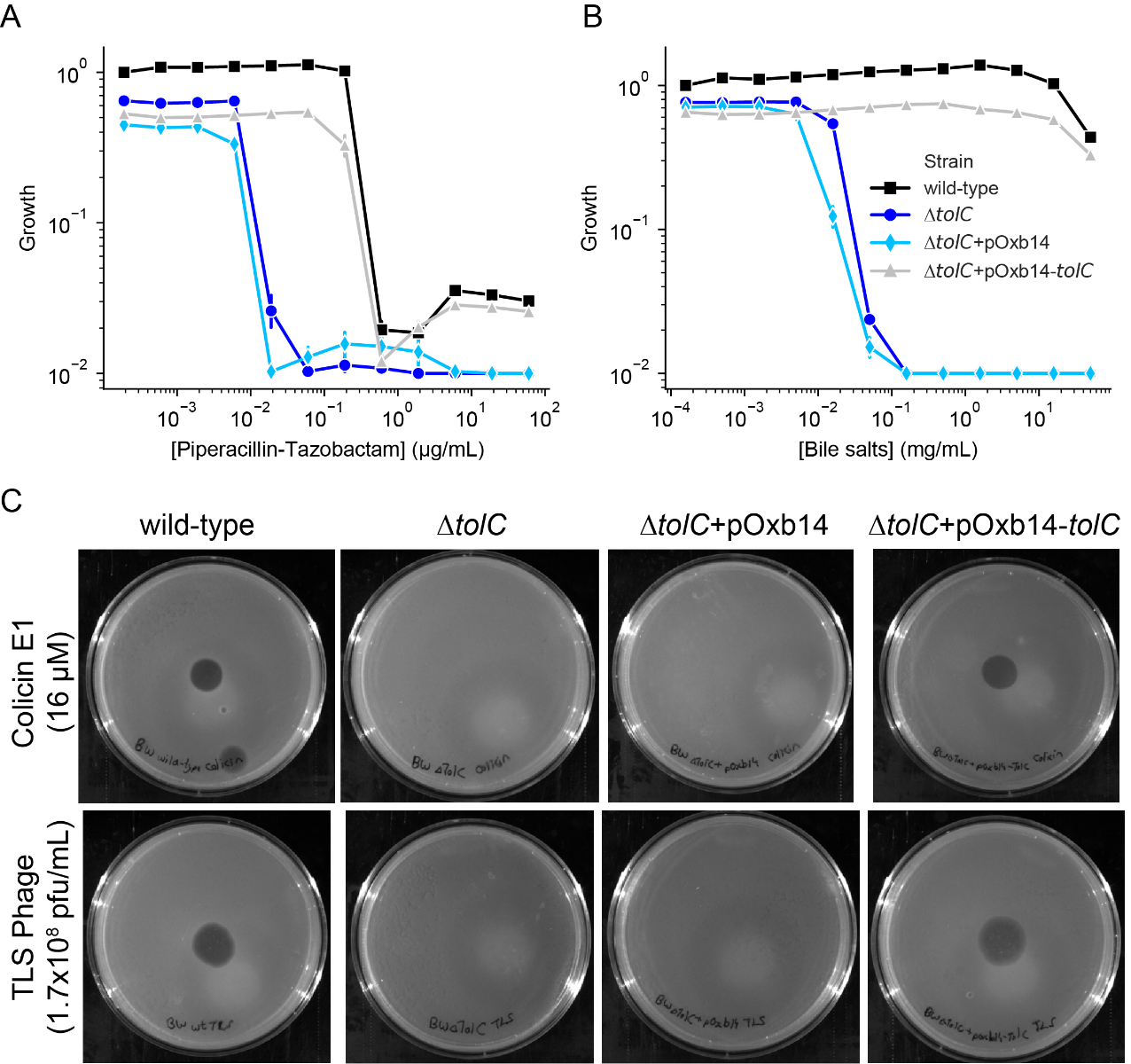


**Figure S2. Supplementing the *tolC* gene into the *E. coli-∆tolC* rescues antibiotic and bile salt resistance.** Dose response curves are reported under piperacillin-tazobactam **(A)** and bile salts **(B)** selection for wild-type BW25113 (black line with square markers), ∆*tolC* (blue line with circle markers), ∆*tolC*+pOxb14 (grey line with triangle markers) and ∆*tolC*+p*tolC* (cyan line with diamonds markers) strains. Growth of the strains are measured at 600nm every 30 minutes. Areas under the growth curves are calculated for each antibiotic and bile salts concentration. Growth values are reported on y-axis after growth values are normalized using wild-type growth in the absence of selection. **(C)** 10µL drop of colicin E1 (16 µM) and TLS phage (1.7x10^8^S) added on top of bacterial lawn of wild-type BW25113, ∆*tolC*, ∆*tolC*+pOxb14 and ∆*tolC*+p*tolC* strains.


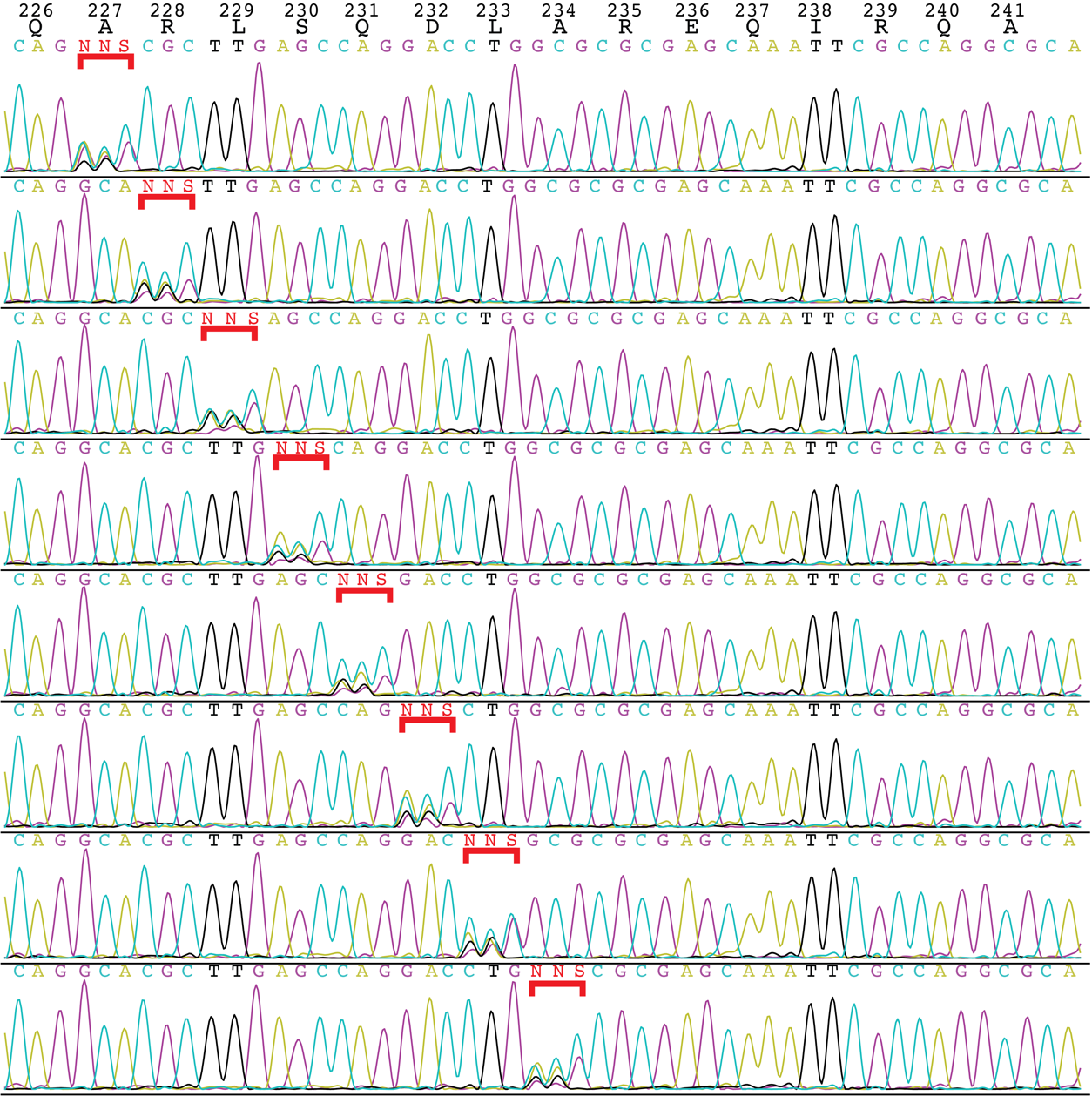


**Figure S3. Confirmation of saturation gene mutagenesis with Sanger sequencing.** Native amino acids for mutated positions (residues 227-234) are shown in black in the top row. Randomization of the intended residue was done separately for each position. Chromatograms demonstrate successful randomization of each codon to NNS (any nucleotide in first two position and either G or C in the third position). In chromatograms, Cyan color was used for Cytosine, Black for Thymidine, Magenta for Guanine, and Yellow for Adenine. Sequence of each fragment is shown above the chromatogram. Randomized regions are highlighted using a red squared bracket.

**
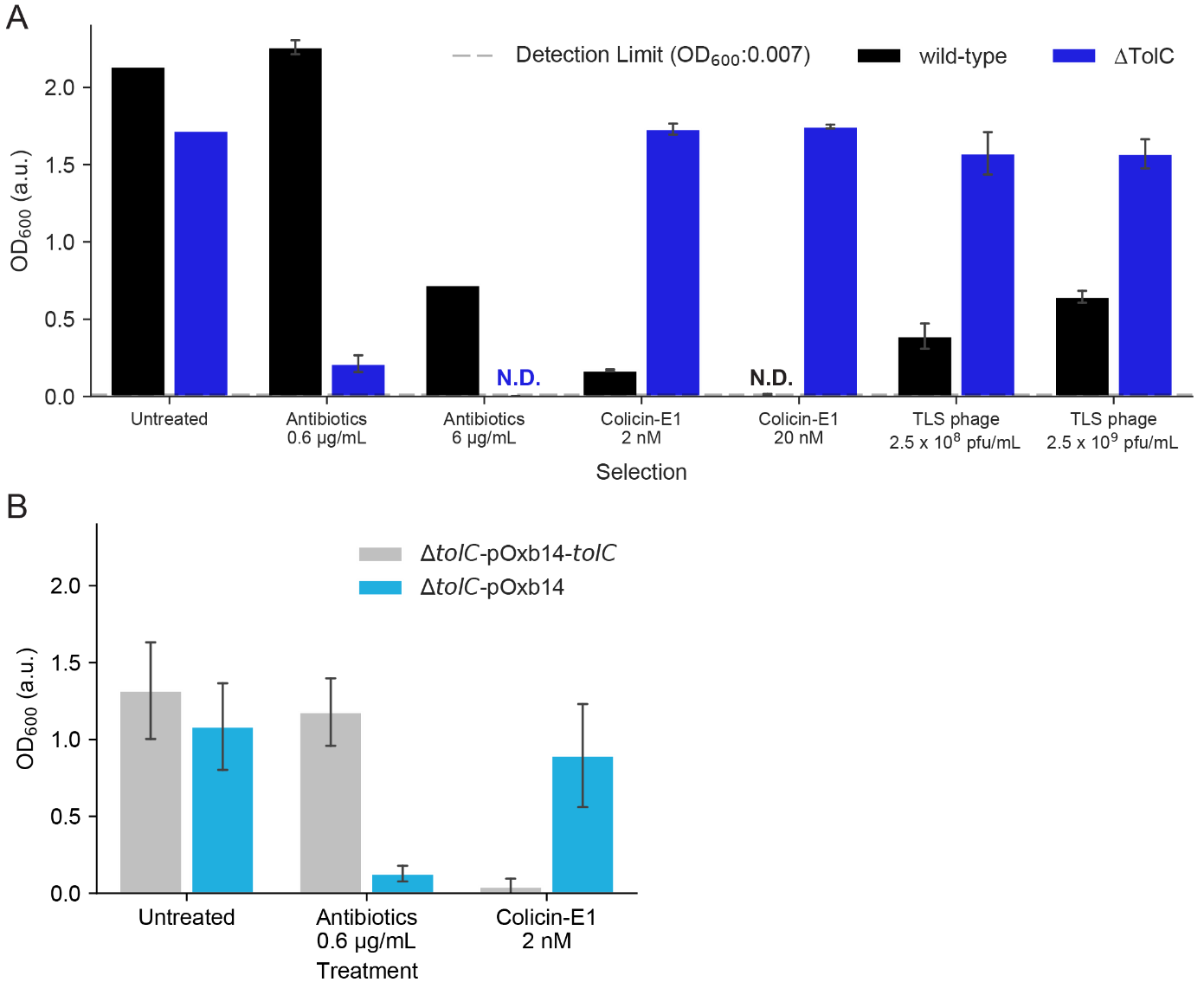
**

**Figure S4. Effects of antibiotic, colicin-E1 and TLS phage selections are quantified with an optical density based assay for wild-type *E. coli* (green) and *E. coli*:∆*tolC* (red).** We plotted cell densities with and without selection. Error bars represent standard deviations. **(A)** Growth values at two concentrations of piperacillin-tazobactam, colicin-E1 and TLS phage were tested. No growth has been observed for ∆*tolC* strain in Piperacillin-tazobactam (6 µg/mL) selection and for wild-type strain for colicin-E1 (20nM) selection which are represented as not determined (N.D. with respective colors). Detection limit for spectrophotometer plotted as horizontal gray dotted line. **(B)** Growth is measured in the presence of piperacillin-tazobactam (piperacillin-tazobactam) and colicin-E1 for the *E. coli*:∆*tolC* strain supplemented with an empty plasmid (cyan) and with a plasmid carrying the wild type *tolC* gene (light gray).

**
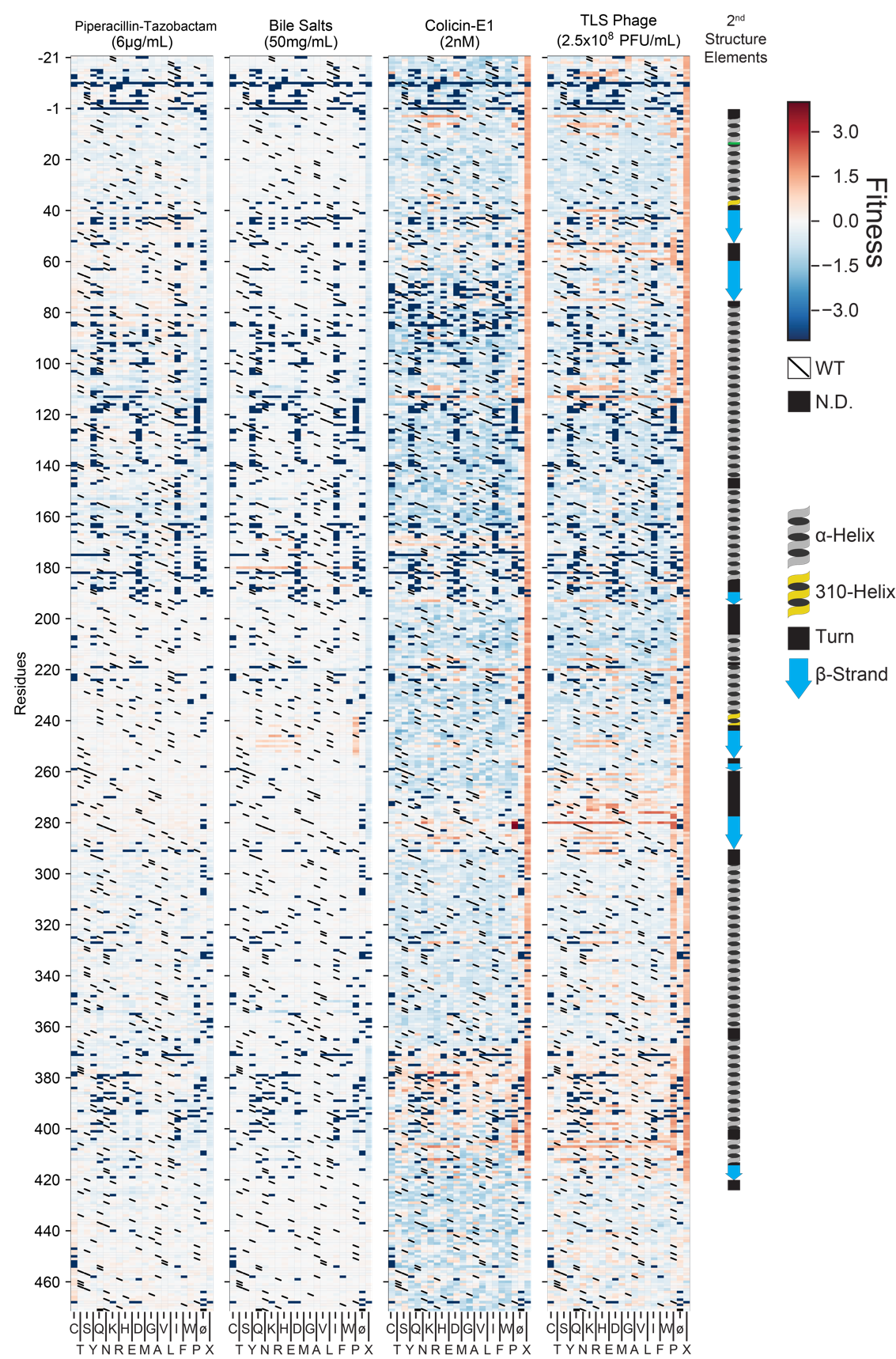
**

**Figure S5. Fitness effects under four different stress conditions are plotted as heatmaps.** Y axis in the heatmaps show the residues. Columns in heatmaps represents synonymous(ø), nonsynonymous and stop codon (X) mutations on each residue. Known secondary structure elements of TolC protein are highlighted in color.

**
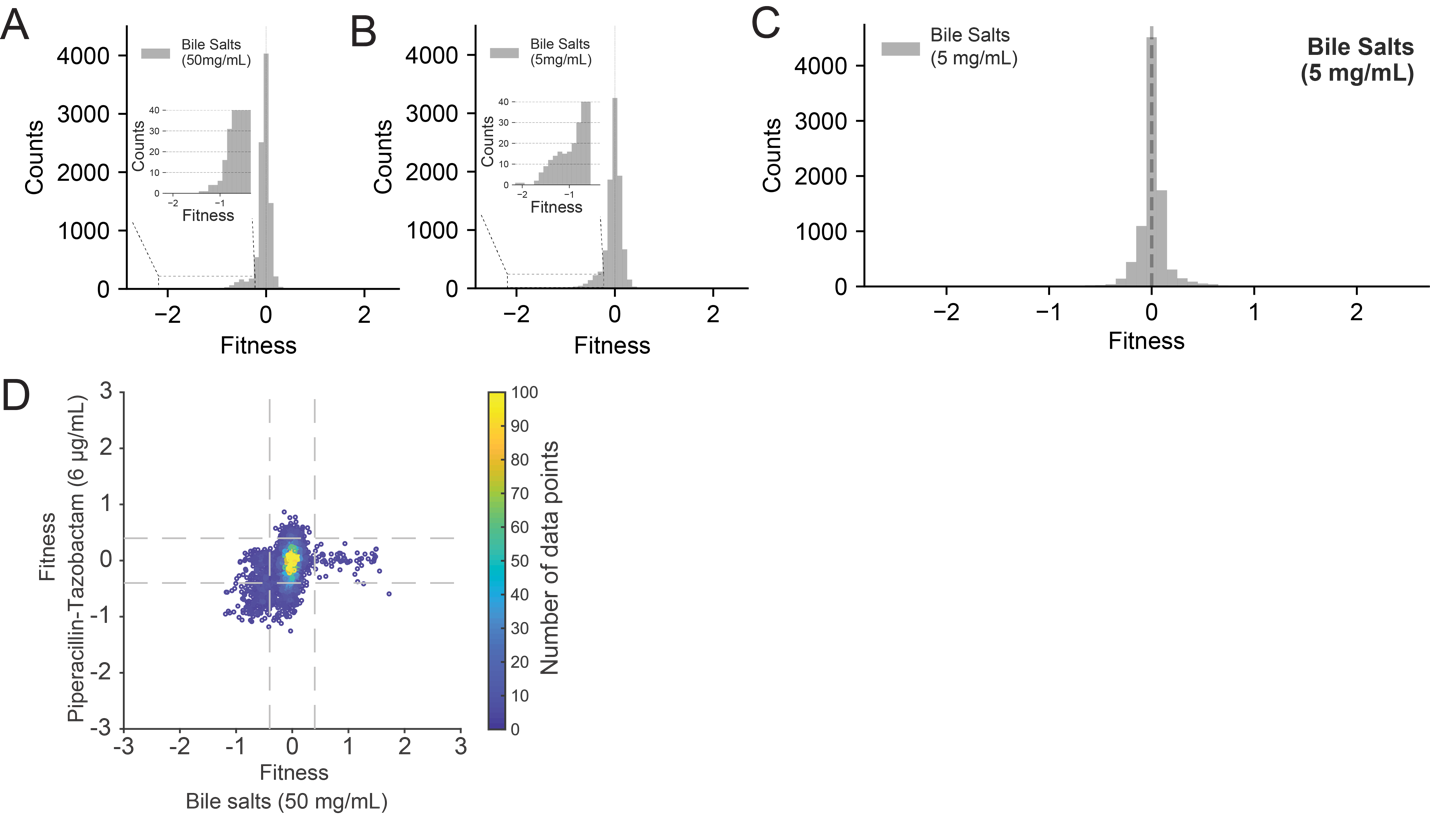
**

**Figure S6: Distribution of fitness effects (DFEs) for bile salts selection.** **(A-B)** DFEs were calculated under two different selection strengths. DFEs under bile salts selection are narrow and centered around neutrality (s = 0) regardless of the selection strength, with tails extending to the left (increased sensitivity, insets). (C) DFE of bile salts selection measured in Illumina NovaSeq platform. Distribution is narrow and centered around neutrality as well. (D) Comparison of fitness effects of TolC mutations under piperacillin-tazobactam (6µg/mL) and bile salts (50 mg/mL) selections. There is a weak correlation in fitness values under these selection conditions (𝝆= 0.28 and p<0.001 Pearson Correlation). Vertical and horizontal dashed lines represent three standard deviation from mean (±0.4).

**
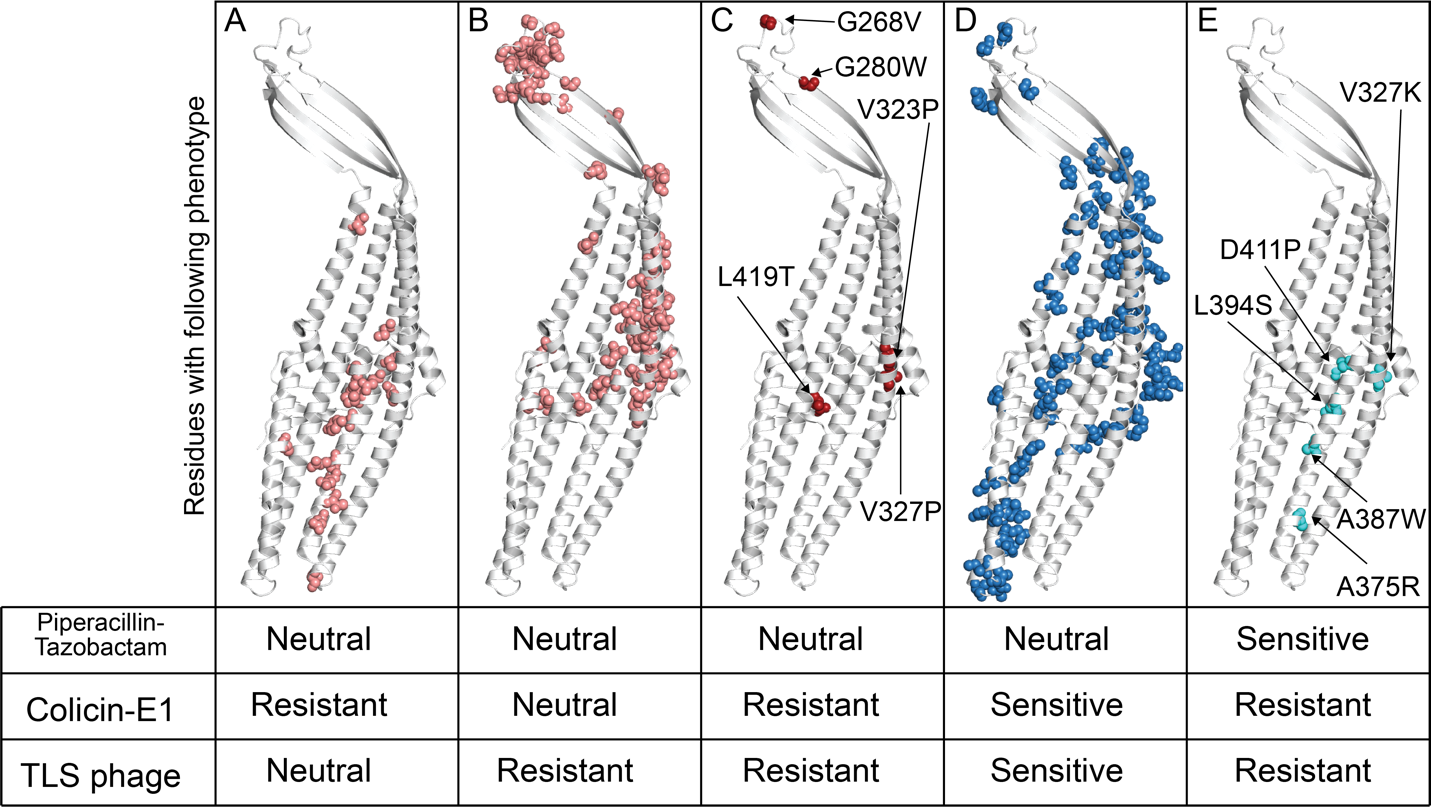
**

**Figure S7: Mutated residues that caused significant fitness changes under at least one of the three selection factors are highlighted in color on monomeric TolC structure.** (Table S2) **(A)** Mutations in pink colored residues confer resistance to colicin-E1 without causing significant fitness changes under other selection conditions. **(B)** Mutations in pink colored residues confer resistance to TLS phage without causing significant fitness changes under other selection conditions. **(C)** Mutations in red colored residues increase resistance to both colicin-E1 and TLS phage without disrupting efflux of piperacillin-tazobactam. **(D)** Mutations in blue colored residues increase sensitivity to both colicin-E1 and TLS phage without disrupting efflux of piperacillin-tazobactam. **(E)** Mutations in cyan colored residues increase resistance to both colicin-E1 and TLS phage and disrupt efflux of piperacillin-tazobactam. These mutations likely cause misfolding of TolC or blockage of the TolC channel as their fitness effects are reminiscent of the loss of the *tolC* gene.

**Table S1:** Mean and standard deviation values for DFEs under all selection conditions: piperacillin-tazobactam (0.6 and 6 µg/mL), bile salts (5 and 50 mg/mL), colicin-E1 (2 and 20nM), and TLS phage (2.5x10^8^ and 2.5x10^9^ pfu/mL).

**Table S2:** Fitness values of mutations represented in Figure S7 are tabulated. There are six sheets in the excel file. First sheet shows the consistent data that has similar fitness values in two different experiments. Remaining five sheets show fitness values for the mutations represented in Figure S7A-E.
